# Supplementary material for: Genetic mapping for agronomic, nutritional, and leaf vein traits in the indigenous crop Gynandropsis gynandra
Source: NPJ Sustain Agric. 2025 Jun 6;3(1):33. doi: 10.1038/s44264-025-00074-0 (PMC12143976; doi:10.1038/s44264-025-00074-0)
Supplement: Supplementary file 1 — Supplementary Information [file 44264_2025_74_MOESM1_ESM.pdf]

Supplementary Information

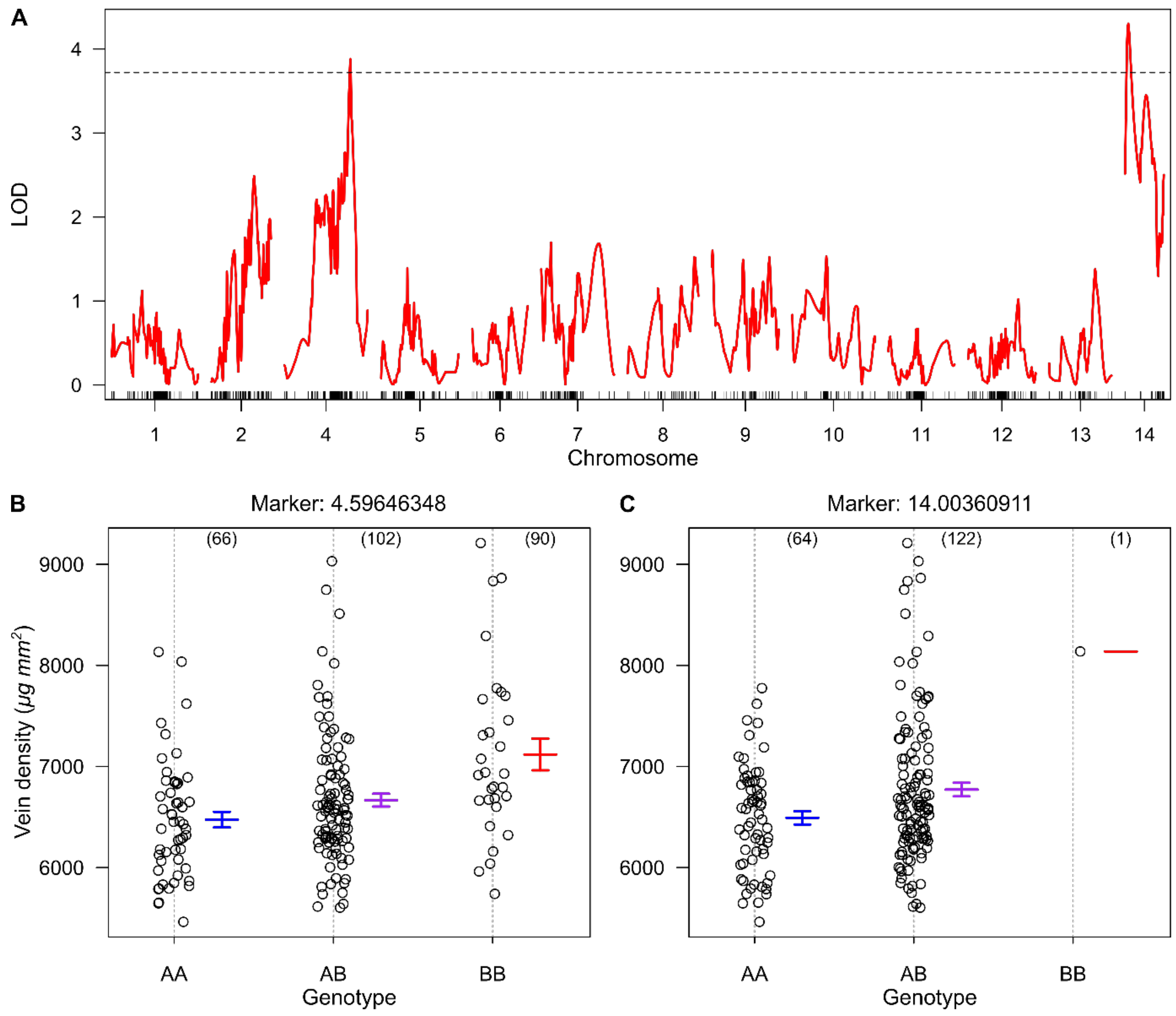

Figure S1: pQTL associated with vein density. (A) Single scan of vein density in the Wag19 population with outliers included. **(B)** The genotypic effect of the nearest marker (4.59646348) to the pQTL *pVd1q* and **(C)** the nearest marker (14.00360911) to *pVd2q*. Numbers in parentheses are the number of individuals with each genotype. Confidence intervals for average phenotypes within each genotype group are shown as blue for AA, purple for AB, and red for BB. Marker names describe physical location; “Chromosome.position (bp)”

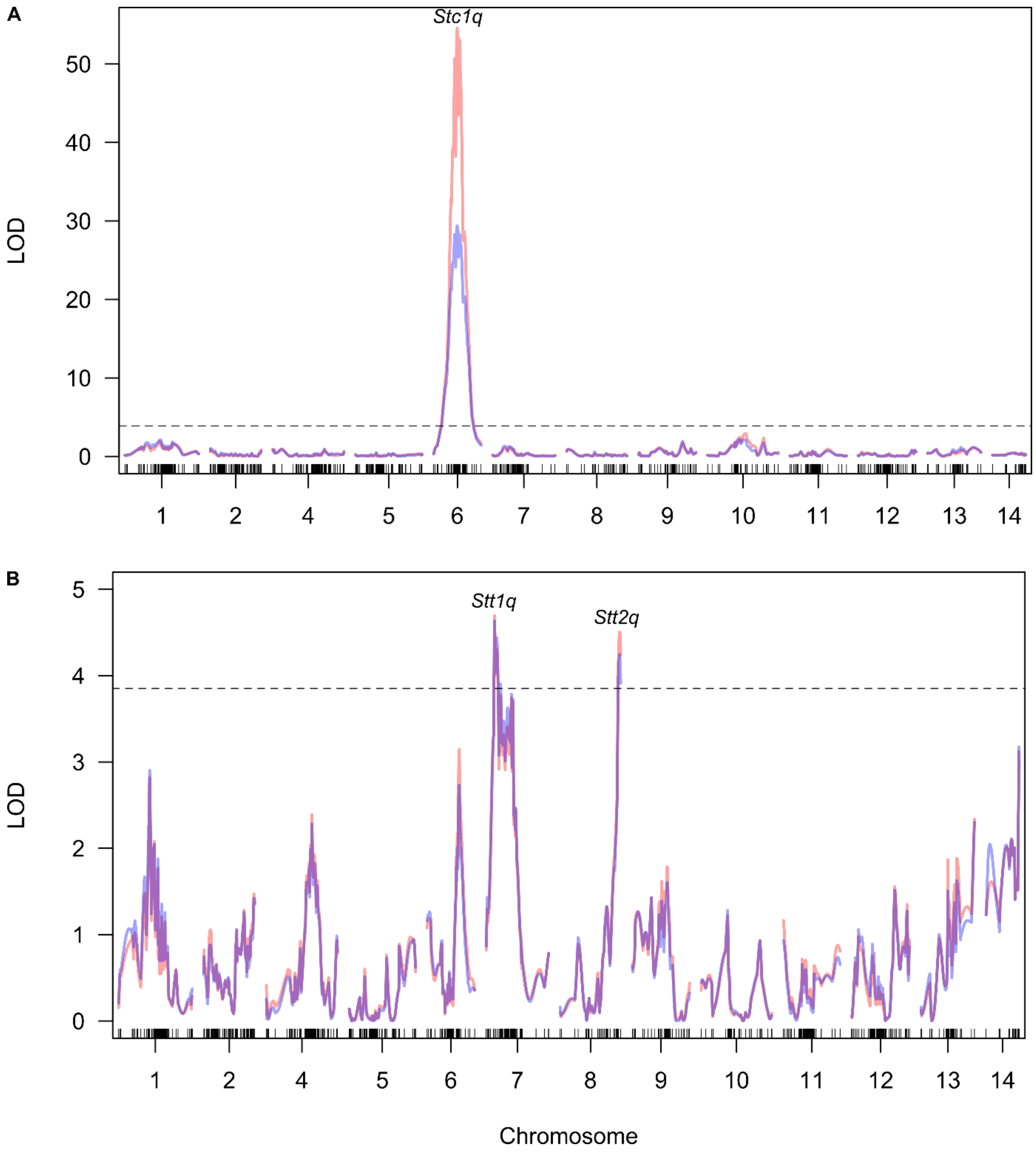

Figure S2: Single QTL scans for (A) stem colour and (B) stem trichome density in Wag19. Red represents the normal model, and blue is the non-parametric model. The dashed line highlights the 0.05 significance threshold after 1000 permutations. The QTL positions are labelled.

Supplementary Tables

Table S1: All assessed agronomic traits were significantly different between the parents of the Wag18 population.

	Malaysia-03			Malawi-02					
**Trait**	**n**	**Mean**	**S.D**	**n**	**Mean**	**SD**	**Test**	**Test stat.**	**P**
Violaxanthin Content	6	4.94	1.01	6	12.11	2.00	Parametric	7.23	<0.001***
Lutein Content	6	21.00	1.36	6	30.30	1.83	Parametric	9.36	<0.001***
Beta-carotene Content	6	50.03	3.81	6	63.56	5.89	Parametric	4.41	0.0017**
Total Carotenoid Content	6	75.97	4.81	6	106.00	5.97	Parametric	9.03	<0.001***
Alpha-tocopherol Content	6	61.54	9.24	6	35.30	9.87	Parametric	-4.52	0.0015**
Flowering Time	6	33.33	2.16	7	43.00	1.91	Non-parametric	42.00	0.0025**
Plant Height	6	106.35	5.60	7	179.23	4.68	Parametric	25.61	<0.001***
Leaf Area	6	40.02	7.05	7	52.38	8.09	Parametric	2.91	0.014*

n = number of replicates, P = significance value, S.D = Standard Deviation, BSCW = Bundle Sheath Cell Width. Significance is indicated by asterixis. Test stat. = Test statistic and is reported as the T or W value for parametric tests and non-parametric tests respectively.

Table S2: Heritability and variance components for traits in each mapping population

Trait	Wag18					Wag19				
	**n**	**H^2**	**V_g**	**V_e**	**V_p**	**n**	**H^2**	**V_g**	**V_e**	**V_p**
VC	199	0.50	2.68	2.68	5.37	168	0.65	9.61	5.14	14.75
LC	199	0.87	17.61	2.69	20.30	168	0.86	70.72	11.48	82.20
β-CC	199	0.81	112.11	25.68	137.79	168	0.76	317.00	97.95	414.95
TC	199	0.89	251.81	30.09	281.89	168	0.78	818.57	229.20	1047.77
α-T**	199	0.76	0.20	0.06	0.26	168	0.50	0.16	0.16	0.32
VD*						187	0.64	9.14	5.14	14.28
BSSW						183	0.54	63.54	54.00	117.54
BSCL						182	0.19	309.35	1305.72	1615.08
FT*	198	0.85	24.00	4.12	28.12	179	0.63	0.0084	0.0049	0.0133
PH*	198	0.97	987.40	26.21	1013.62	182	0.49	673232.7	709954.7	1383187
LA**	189	0.90	0.25	0.03	0.28	179	0.58	1.17	0.84	2.02

n = number of F_2 lines phenotyped, H^2 = Broad-sense Heritability, V_g = Genetic Variance, V_e = Environmental Variance, V_p = Phenotypic Variance, BSW = Bundle Width, FT = Flowering Time, PH = Plant Height, LA = Leaf Area, LW = Leaf Weight, SC = Stem Colour, ST = Stem Trichome density. *Estimates carried out on Wag19 transformed data; **estimates carried out on Wag18 and Wag19 transformed data.

Table S3: Summary of linkage map metrics for Wag18 and Wag19 mapping populations

Metric	Wag18	Wag19
Number of individuals	206	187
Number of markers	297	920
Total map length (cM)	984.87	1645.02
Average chromosome length (cM)	82.07	126.54
Total crossovers	3293	5465
Recombination rate	1.33	2.35
Genotyping error rate	~0	~0
Mapped chromosomes	1, 2, 4, 5, 6, 7, 8, 9, 10, 11, 12, 13	1, 2, 4, 5, 6, 7, 8, 9, 10, 11, 12, 13, 14

Recombination rate is in terms number of cross overs per individual per chromosome.

Table S5: Kranz traits, alpha-tocopherol content, flowering time, leaf area, leaf weight, trichome density and stem colour scores were significantly different between the parents while carotene content and plant height were not in the Wag19 population.

	Malaysia-01			Malawi-01					
**Trait**	**n**	**Mean**	**S.D**	**n**	**Mean**	**SD**	**Test**	**Test stat.**	**P**
Violaxanthin Content	5	13.06	1.62	5	16.17	2.99	Parametric	1.80	0.13
Lutein Content	5	31.33	3.93	5	37.15	2.34	Parametric	2.25	0.07
Beta-carotene Content	5	73.56	11.37	5	77.65	7.13	Parametric	0.54	0.61
Total Carotenoid Content	5	117.94	16.69	5	130.97	12.45	Parametric	1.28	0.31
Alpha-tocopherol Content	5	21.71	8.24	5	8.63	2.82	Parametric	-2.58	0.049*
Vein Density	5	5713	267	5	9822	536	Parametric	15.35	<0.001***
BSSW	4	108.76	8.48	5	88.71	6.37	Parametric	-4.07	0.0048**
BSCL	4	44.51	6.55	5	33.31	5.60	Parametric	-2.77	0.028*
Flowering Time	5	38.20	2.68	5	52.20	2.39	Non-parametric	25	0.011*
Plant Height	5	39.60	3.65	5	45.60	13.01	Parametric	0.99	0.37
Leaf Area	5	18.38	1.83	5	59.57	19.77	Parametric	4.64	0.0094**
Stem Trichome Density	5	0.40	0.54	5	1.8	0.44	Non-parametric	24	0.15*
Stem Colour	5	0.20	0.45	5	1.6	0.54	Non-parametric	24	0.15*

n = number of replicates, P = significance value, S.D = Standard Deviation, BSCW = Bundle Sheath Cell Width. Significance is indicated by asterixis. Test stat. = Test statistic and is reported as the T or W value for parametric tests and non-parametric tests respectively.

Table S4: Overall summary of putative QTL identified in the Wag18 population

Phenotype	pQTL number	pQTL name	Nearest marker (chr.bp)	Additive effect	Dominance effect	LOD	Model formula	Model LOD	PVE by model (%)
Violaxanthin content	Q1	*pVio2q*	1.05951559	-2.78	0.83	4.98	y ~ Q1 + Q2	7.57	16.07
Violaxanthin content	Q2	*pVio3q*	1.13366757	2.89	0.05	6.28	y ~ Q1 + Q2	7.57	16.07
Lutein content	Q1	*pLut1q*	2.2745371	-2.08	0.01	4.64	y ~ Q1	4.64	10.23
Alpha-tocopherol content*	Q1	*pAtc1q*	9.00780456	-0.42	0.10	17.41	y ~ Q1	17.41	33.16
Flowering time	Q1	*pFlt1q*	6.36025417	3.00	-2.02	9.19	y ~ Q1	9.19	19.24
Plant height	Q1	*pSiz1q*	1.13687113	25.12	-1.30	12.38	y ~ Q1	12.38	25.02
Leaf area*	Q1	*pSiz1q*	1.11928029	0.38	-0.11	8.33	y ~ Q1	8.33	18.38

PVE = Percentage of Variance Explained. Nearest marker name is based on physical position: "Chromsome.position (bp)". Negative and positive additive effects mean the allele from Malaysia-03 and Malawi-02 respectively is responsible for an increase in the trait. *Analysis carried out on log transformed data.

Table S6: Overall summary of putative QTL identified in the Wag19 population

Phenotype	*p*QTL number	pQTL name	Nearest marker (chr.bp)	Additive effect	Dominance effect	LOD	Model formula	Model LOD	PVE by model (%)
Violaxanthin Content	Q1	*pVio1q*	10.03027582	1.07	-2.20	4.77	y ~ Q1	4.77	12.26
Alpha-tocopherol content*	Q1	*pAtc2q*	11.41198362	-0.23	-0.20	5.56	y ~ Q1	5.56	14.14
Vein Density****	Q1	*pVd1q*	4.59646348	1.50	-0.75	3.88	y ~ Q1 + Q2	5.15	12.26
Vein Density****	Q2	*pVd2q*	14.00360911	4.84	-3.70	4.30	y ~ Q1 + Q2	5.15	12.26
Flowering time**	Q1	*pFlt2q*	6.46920704	0.083	-0.033	10.85	y ~ Q1	10.85	24.35
Plant height***	Q1	*pSiz2q*	1.17961377	514.58	419.87	7.46	y ~ Q1 + Q2	11.62	25.47
Plant height***	Q2	*pHt1q*	2.38898522	-528.14	12.25	4.48	y ~ Q1 + Q2	11.62	25.47
Leaf area****	Q2	*pLa1q*	1.70602349	-0.27	0.99	4.98	y ~ Q1 + Q2 + Q3	14.51	31.15
Leaf area****	Q1	*pSiz2q*	1.18700207	0.53	0.28	4.59	y ~ Q1 + Q2 + Q3	14.51	31.15
Leaf area****	Q3	*pLa2q*	12.38068542	0.73	-0.19	5.75	y ~ Q1 + Q2 + Q3	14.51	31.15
Stem trichomes	Q1	*pStt1q*	7.00721601	-0.26	-0.08	5.18	y ~ Q1 + Q2	9.67	21.71
Stem trichomes	Q2	*pStt2q*	8.44525256	0.25	0.15	4.98	y ~ Q1 + Q2	9.67	21.71
Stem colour	Q1	*pStc1q*	6.30828142	0.77	0.81	62.67	y ~ Q1 + Q2 + Q1:Q2	62.75	79.56
Stem colour	Q2	*pStc2q*	7.00205393	-0.076	-0.037	8.17	y ~ Q1 + Q2 + Q1:Q2	62.75	79.56
Stem colour	Q1:Q2					6.93	y ~ Q1 + Q2 + Q1:Q2	62.75	79.56

PVE = Percentage of Variance Explained. Nearest marker name is based on physical position: “Chromsome.position (bp)”. Negative and positive additive effects mean the allele from Malaysia-01 and Malawi-01 respectively is responsible for an increase in the trait. Analysis carried out on *log, **cube-root, ***squared, ****square-root transformed data.

Table S7: Summary of ordinal regression on ordinal traits in the Wag19 population

Trait	pQ1	pQ2	Model	AIC	pQ1 L t.value	pQ1 Qu t.value	pQ1 L p.value	pQ1 Qu p.value	pQ2 L t.value	pQ2 Qu t.value	pQ2 L p.value	pQ2 Qu p.value
Stem trichomes	*pStt1q*	*pStt2q*	y ~ Q1	305.28	-4.25	0.34	<0.001	0.73				
Stem trichomes	*pStt1q*	*pStt2q*	y ~ Q2	299.61					4.03	-1.02	<0.001	0.31
Stem trichomes	*pStt1q*	*pStt2q*	y ~ Q1 + Q2	274.65	-4.42	0.76	<0.001	0.45	3.99	-1.73	<0.001	0.085
Stem colour	*pStc1q*	*pStc2q*	y ~ Q1	161.57	5.91	-5.24	<0.001	<0.001				
Stem colour	*pStc1q*	*pStc2q*	y ~ Q2	310.21					0.26	0.23	0.80	0.82
Stem colour	*pStc1q*	*pStc2q*	y ~ Q1 + Q2	160.91	6.09	-5.32	<0.001	<0.001	-1.82	0.87	0.070	0.39

pQ = pQTL; L = Linear; Qu = Quadratic. pQTL name is based on linkage map position: “pChromosome:position(cM)”. Negative and positive Linear t.values mean the allele from Malaysia-01 and Malawi-01 respectively is responsible for an increase in the trait. Negative and positive Quadratic t.values indicate dominant effects.
